# Introduction: Contesting the moral worlds, scales, and epistemics of energy transitions

**DOI:** 10.1177/0308275X241269650

**Published:** 2024-09-15

**Authors:** Zeynep Oguz, Mark Goodale

**Affiliations:** University of Edinburgh, UK; 27213 Laboratory of Cultural and Social Anthropology (LACS), University of Lausanne, Switzerland

**Keywords:** Anthropology of energy, climate justice, energy transition, just transitions, moral worlds, renewable energy, scale-making

## Abstract

The introduction to this special issue, *Contesting Transitions: New Directions in the Anthropology of Energy, Climate Justice, and Resource Imaginaries*, takes stock of the current state of debate within anthropology and allied fields over the contradictions, slippages, and inequalities at the centre of the global energy transition. Across a wide range of critical case studies, the contributions underscore the importance of attending to what is being elided by dominant discourses and forms of production, such as alternatives to socio-material understandings of energy and resistance to the inevitability of extractivism as the basis for new ways of living. Even more, the collection takes up and problematizes the concept of ‘transition’ itself on historical, ethnographic, and epistemological grounds. After describing the themes that emerge from the special issue, and explaining how these themes point toward new configurations of research, theory-building, and critical intervention, the introduction concludes with a broader argument about the indispensable place of a critical anthropology in debates over energy and Anthropocenic harm.

‘We have to make drastic reductions in greenhouse gas emissions and accelerate the transition to renewable energy sources’, declared the general secretary of the World Meteorological Organization (WMO) in 2024 (*TIME*, 2023). As we were writing our introduction to this special issue on new directions in the anthropology of energy, climate justice, and resource imaginaries, a massive heatwave was expected to hit Europe in July during a year that was shaping up to either match, or surpass, 2023 as the hottest year on record (Carbon Brief, 2024). The UK alone had already experienced its warmest May and spring on record (*The Guardian*, 2024). It wasn’t all bad news, however. The International Energy Agency reported that the amount of renewable energy capacity added to global energy systems grew by 50% in 2023, reaching almost 510 gigawatts (GW), with photovoltaics, or solar panels, accounting for three-quarters of these additions (IEA, 2024).

Despite such seemingly hopeful prospects, however, a recent *TIME* magazine article cautioned its readers that the world needed to act faster, citing an article on climate change in the journal *Nature* that found that:

If humanity wants to have a 50-50 chance of limiting global warming to 1.5°C, that means maximum emissions of another 250 billion metric tons of CO_2_, effectively giving the world just over six more years of business-as-usual before it must hit net-zero emissions. (*TIME*, 2023)

As the earth scientists implored, once again, the world needed to make much more rapid advances in its technological transition towards ‘renewable’ energy sources.

Despite such calls for urgent technological mobilization, energy transitions are being increasingly contested on a number of different political, institutional, and theoretical grounds. In both the global North and global South, from right- to left-wing political orientations, there is a growing backlash against renewable energy and energy transition policies. Progressive environmental movements and local communities have critiqued the extent to which the so-called green energy transition continues to rely on the extraction of non-renewable resources, often under conditions associated with grave human rights abuses and environmental racism.

At the same time, a right-wing ‘greenlash’ has gained momentum in many places, fuelled by conservative forces and exacerbated by rising costs of living and geopolitical conflict. In such a context, policy experts and academics alike suggest that ‘climate justice’ should be prioritized in energy transitions, rather than the technological process of energy transition itself. As one climate activist puts it, ‘Rather than focusing solely on abstract emissions targets, we should underscore the local, immediate, and long-term benefits of sustainable policies’ (Leyi, 2024).

However, the very meaning of ‘climate justice’ itself is contested, in addition to who should be its main beneficiaries. Is everyone equally deserving of climate justice in this era of Anthropocenic deterioration, in which the generalizing frame of ‘human impact’ obscures centuries of unequal responsibility? Even more, most of the basic concepts in the field are also contested, including ‘transition’ and ‘renewable’, beyond the social and political questions about the capacity of energy transitions to address broader structural and historical patterns of injustice.

Such multi-layered forms of contestation reveal that the energy transition is much more than a global technological challenge: it is both an actual and imaginary shift with wide-ranging political, social, ethical, and affective consequences. In this context, anthropology is well positioned to examine these consequences across the range of the discipline’s methodological and theoretical approaches. As a contribution to this collective endeavour, this special issue of *Critique of Anthropology* builds on and extends a growing body of work in anthropology and related fields – such as political ecology and science and technology studies (STS) – that has examined empirically and critically the cultures and epistemologies of energy; the everyday constitution of expert knowledge and infrastructures; and the intersection of other forms of power and meaning with energy projects and materialities; among others (see Strauss et al., 2013; Wilson et al., 2017).

At the same time, the special issue also contributes to debates around the temporalities and relationalities of energy transitions. People are being increasingly called upon to put their faith in new energy technologies in order to accelerate the process of transition in ways that are blurring the boundaries between human and technological agency. As one group of scholars has put it, the idea that ‘technology-led transitions’ will lead to “societal benefits” [is] based on unrealistic understandings of people and our relationships with technology and are therefore at great risk of failure’ (Pink et al., 2023: 4).

This special issue is the result of a three-day international workshop on the anthropology of energy held at the University of Lausanne in June 2023. The workshop emerged from our sense that the anthropology of energy should expand beyond its existing focuses on the cultural, political and socio-material dimensions of energy organization and practice. Anthropologists are trained to render the ‘familiar’ unfamiliar (and vice versa) by revealing the quotidian and pragmatic operations of meaning and knowledge systems, to consider ethnographically how people who are closest to these operations – workers, miners, CEOs, intermediaries, experts, and so on – evaluate energy and energy transitions. In so doing, an anthropological lens can denaturalize the very terms of the debate through which energy transitions are supposed to take place. In this regard, the diverse contributions presented at the 2023 workshop in Lausanne sought to develop a denaturalized (and denaturalizing) and plural (and pluralizing) understanding of energy transitions, an approach that participants brought to the conceptual and empirical understandings of ‘energy’ and ‘transition’ themselves.

Beyond the anthropological unpacking of the keywords and concepts at the heart of the long-term shift from the age of oil to the ages of wind, solar, and lithium, *Contesting Transitions* also examines how anthropological framings can better illuminate the challenges of energy transitions, not just by analysing the elements that are contested but also by critically attending to the often taken-for-granted and invisible terms of the debate, for example, around numbers and metrics, broader moral discourses, and the implicit practices of scale-making that structure the presentation of energy systems by policy makers.

To this end, the articles in this special issue, taken together, reveal the ways in which anthropologists approach a number of fundamental questions. What kinds of divergent meanings do people attach to energy and energy transitions as categories of practice and complex socio-material configurations? How are concepts like ‘green’, ‘clean’ and ‘renewable’ understood and lived by people in relation to existing political and ideological imperatives? What kinds of new social formations, modes of subjectivity and norms emerge in relation to energy transitions, as both strategies of mediation and frameworks for resistance? How are energy transitions naturalized in ways that exclude other, more radical, propositions for responding to the ongoing legacies of Anthropocenic harm? And, finally, in what ways can anthropological forms of knowledge and practice themselves constitute categories of both contestation and energetic future-making?

## Anthropologies of energy: Injustice, futures, and actually existing transitions

Before introducing the different themes and articles of the special issue, it is important to provide a short overview of existing directions in the anthropology of energy. Scholars have convincingly demonstrated the ways in which energy transitions are inherently political, or even a mode of politics (Boyer, 2014). Ethnographies of power (Loloum et al., 2021), in this respect, highlight both the political and energetic dimensions of phenomena. Building on Foucault’s concept of biopower (the management of life and population), Dominic Boyer proposes an ‘alternative genealogy of modern power’ (Boyer, 2014: 325), arguing that: ‘There could have been no consolidation of any regime of modern biopower without the parallel securitization of energy provision and synchronization of energy discourse’ (2014: 327).

Other scholars emphasize the ways in which energy and energy transitions are interwoven into social worlds marked by inequality and histories of injustice. For example, Leo Coleman (2021) argues that an anthropology of energy should go:

beyond energopolitics, [to focus] less on the global organization of energetic flows or the reprogramming of work as ‘energy’ in carbon modernity, and more on the energy of the abstract ideas, solidarities and immaterial bonds (of obligation and reciprocity and belief) that link people to each other and to things. (Coleman, 2021: 189)

Relatedly, in a recent special issue on African energy transitions, Michael Degani and colleagues (2020) ask:

What sort of social and political formations find purchase in the technical and material affordances of air, sunlight, gas or recycled waste? And what are the stakes for theorizing the politics of energy that does not take fossil fuels as its paradigmatic case but rather begins with a complex field of global energy realignment, comprising multiple sites and sources? (Degani et al., 2020: 2)

Such questions revolve around another contested social fact: ‘the future’. From ideas of renewal, development, and progress (Abram et al., 2022), supposedly sustainable energy projects produce collective hopes about fixing or healing the destruction caused by environmental violence and climate change (Bryant and Knight 2019). These futures are categorically specific in the sense that they suggest ways to harness technological change to affect sociopolitical relations, including historical inequalities (Jasanoff and Kim, 2013). That the projected transformation of social relations has not happened yet, and mostly likely never will, only adds to the fraught nature of energy futures (Goodale, 2023a; Szeman and Barney, 2021). And because energy futures are so dependent on existing capitalist relations of production and the management of global labour, the transition toward these futures can be understood as a process that is both ‘fixed and foreclosed’ (Goodale, 2023b).

As a specific consequence of this kind of foreclosure, the replication of extractive practices in the unfolding energy transition has exacerbated the climate crisis. Site-specific injustices and supply-chain inequities continue to plague renewable energy programmes. Howe (2019a) and Boyer (2019) critique these programmes for reinforcing extractivist politics rather than fostering inclusivity, community consultation, and equitable power and profit distribution. Their research on wind power in Mexico highlights how new energy developments can involve devastating encroachments on Indigenous lands, raising questions about the ways the energy transition is merely reinforcing the kinds of environmental and political violence associated with the preceding decades of petropolitics.

Other scholars have brought a critical ethnographic perspective to bear on the more granular dynamics of energy transitions, which, among other things, underscores the ways in which diverse energy transitions do not merely replicate global patterns but must be understood in their own terms. For example, Andrew Curley (2018) has examined how the Navajo Nation’s attempt to transition from coal to wind and solar energy through the ‘Navajo Green Jobs’ programme mostly succeeded in introducing neoliberal assumptions about governance and development that undermined tribal commitment to new energy technologies. The resulting ‘transition’ overlooked the spatial and social complexities of energy production, focusing instead on local entrepreneurship over-centralized tribal control. In the end, the Navajo Nation rejected the ‘Navajo Green Jobs’ initiative and decided to reinvest in coal production.

And based on her research in Ghana, Gökçe Günel (2021) shows how an increasing volume of rooftop solar panels installed by affluent individuals and institutions in the aftermath of a four-year electricity crisis in the country led to declining participation in the electricity grid and higher electricity rates for everyone who couldn’t afford to install such a relatively expensive technology. In response this growing energy inequality, policy makers in Ghana searched for innovative business models, turning to green loans as a way of expanding the country’s class of solar consumers. As a result, while a select few managed to ‘leapfrog’ to renewables in Ghana, others continued to endure the grid, struggling with unsteady electricity provision and increasing tariffs.

In a context of both persistent and novel injustices associated with energy transitions, Sovacool and his colleagues (Sovacool et al., 2023) call for intersectional approaches to energy and climate justice, incorporating feminist, Indigenous, anti-racist, and postcolonial perspectives to counter the colonial, liberal, utilitarian, and masculinist assumptions at the centre of global energy economics and policy making.

Similarly, Dunlap and Tornel (2023) argue that energy justice frameworks perpetuate images of justice that reinforce Eurocentric and state-centric developmental models, while excluding justice-making projects and perspectives more closely tied to the subversive programmes of climate justice social movements.

And finally, Bell, Daggett, and Labuski (2020) suggest that we analyse energy politics along four intersecting coordinates: the political (democratic, decentralized, and pluralist); the economic (prioritizing human well-being and biodiversity over profit and unlimited growth); the socio-ecological (preferring relationality over individualism); and the technological (privileging distributed and decentralized fuel power and people power). As they argue, a feminist approach is well-suited for ‘navigating the tangled web of power, profit, and technological innovation that comprises human fuel use’, and which carries over into the tangled webs of new energy technologies and practices.

Amid both increasing energy polarization and deepening energy inequality, anthropological approaches provide a penetrating perspective on the political, historical, and place-specific dynamics of energy transitions, one whose scope of vision ranges far beyond technological and determinist understandings. Further, in addition to political economic and ecological dynamics, debates around energy and climate (in)justice are ultimately tied to contested moral worlds, as well as wider struggles over who and what matters: across the different links in the energy supply chain; across practices of scale- and future-making; and across competing forms of knowledge and knowledge claims.

In the following sections, we develop these themes in more detail and show how the articles in this special issue collectively point toward new configurations of research, theory-building, and critical intervention.

## Energy’s moral worlds

People experience, conceptualize, and evaluate energy matters in multiple and varied ways, including how they make ethical judgements about the role of energy in the types of ‘good societies’ they imagine for themselves. The foregrounding of ‘energy ethics’ captures the ways in which people understand and ethically evaluate energy (Smith and High, 2017). It also shines a critical light on the taken-for-granted and moralized assumptions about both carbon-intensive and renewable energies.

Building on this perspective, a number of articles in this special issue broaden this approach by locating the ethics of energy and energy transitions within wider moral worlds, in which people and communities experience energy relations as part of ideological, religious, and ritualized formations.

Mette High’s study of oil and gas industry participants in Colorado shows how contemporary ‘wildcatters’ mobilize a sacred account of energy capitalism to dismiss the green energy transition, which appears to them as a profane affront to cherished values of energy density, innovation, and engineering realism. As she argues, the moral worlds of petromodernity are conceived by her interlocuters in stark contrast to the imagined (im)moral worlds of renewable energy, which are viewed as political, rather than scientific, technologies.

In a quite different context of competing moral worlds, Pauline Destrée’s article examines the ways in which energy policy makers in Ghana invest existing fossil fuels with different moral registers, partly as a response to what are viewed as the neocolonial pressures of global climate mandates, and also partly in order to justify continuing national energy development. In arguing that gas has a more morally sustainable valence than oil as an energy source, one that, moreover, forms a ‘bridge’ to more renewable technologies, Ghanaian politicians and petro-capitalists forge a ‘moral frontier’ that reshapes what political and moral possibilities can be considered as a challenge to wider energy inequalities.

Michael Degani’s article considers a moral frontier of a different kind, one that is constructed both in relation to, but also critically apart from, the Tanzanian state. His study of electricity microgrids – which provide electricity in the wide provisioning gaps between the national grid and household energy generation – focuses on religious missions, abbeys, and convents, which have a long history in Tanzania of operating ‘run-of-the-river’ power stations and other local and regional off-grid systems. As he argues, the moral worlds of energy in rural Tanzania can’t be understood apart from wider spiritual economies and moral infrastructures.

And Anna Szolucha’s research in northern England at the height of public controversy over shale gas exploration – which depends on a technology widely referred to as ‘fracking’ – adds yet another dimension to our understanding of the moral worlds of energy transitions. Drawing from anthropological theory on religious practice, she shows how public planning sessions became secular ritualized spaces in which communities opposed to the government’s proposals for shale gas development experienced a kind of ecstatic transformation, despite the efforts of government officials to frame planning sessions as bureaucratic and technocratic exercises.

## Places, scales, and histories of energy

Beyond grappling with the competing moral worlds of energy transition in local and global contexts, articles in the special issue also interrogate the ways in which energy processes produce, and are produced by, places, scales, and sedimented histories (Hecht, 2018; Liboiron, 2021). Focusing on the embedded and embodied dimensions of energy transitions also leads to wider questions about the tension between global policy making and local and regional futures. As Cymene Howe puts it: ‘Is the betterment of a global climate – through the tools of renewable power – to be prioritized over localized ecological spaces where that energy is produced and conveyed?’ (2019b: 163).

In their article, Jerome Whitington and Zeynep Oguz show how energy histories and futures are entangled in complicated ways in the legacy mining communities of central Appalachia in the United States. Through a process they described as ‘energetic place-making’, more recent solar projects – both commercial and community – must contend with the geosocial imprints of coal extraction, imprints that are expressed in the region’s transformed geology, socioeconomic inequalities, and racialized hierarchies. As they argue, energetic place-making itself becomes a site of contestation, in which some communities embrace solar energy as a way of breaking the ‘chokehold’ of coal mining and its association with post-industrial decay.

Although they don’t use the term, Alison Kenner and her co-authors likewise examine the ethnographic particularities of energetic place-making, in this case in the heart of urban Philadelphia. They examine what they describe as the ‘stubborn infrastructures’ of energy in the city, the ways in which the city’s house- and neighbourhood-level energy architectures encode social vulnerability, utility debt, and decades of municipal policy making. As they argue, a granular ethnographic approach to these ‘local energy ecologies’ undermines the apparent contradictions that are so common in debates over the energy transition, for example, the supposed opposition between affordability and sustainability.

In his article, Mark Goodale shifts the theoretical lens from place-making to the related phenomenon of scale-making, which allows him to explore a number of ‘topographies of production’ that coexist alongside Bolivia’s state-led lithium project. These topographies are populated by communities and economic relations that resist, in different ways, the pressure to reorient social and productive worlds around the extraction of one of the world’s most important ‘critical’ minerals. As he argues, by attending to the ‘inter-scalar frictions’ that mark Bolivia’s fraught lithium industrialization process, we gain a better understanding of how energy futures *also* depend on their antitheses, the counter-futurities that might, or might not, offer what Cymene Howe has described as an ‘antidote to the Anthropocene’ (2019a: 2).

## Energy epistemics and (in)commensurabilities

Scholars have also conducted important research around questions of *who* and *what* matters in energy transitions, including the ways in which only certain kinds of energy futures are privileged in dominant public discourse, a form of inclusion that works in part by excluding other, more radical, alternatives. For example, in their ethnography of the lived experiences of the *Energiewende* in Germany, Morton and Müller (2016) argue that the energy transition from coal to other energy sources is not just a technological shift. Rather, ‘The contest over the future of coal in Lusatia can be seen as a struggle to control key cultural narratives of home, belonging, ecological modernization, climate change, and democratic deficit’ (Morton and Müller, 2016: 277).

However, as the last grouping of articles in this special issue demonstrates, the *how* of energy transitions also matters. How are problems of energy transition conceived, explained, measured, and ‘solved’? And do the openings and closures of particular forms of energy knowledge have implications for the possibilities of energy and climate justice?

Simon Abram’s article brings an anthropological perspective to what she describes as the ‘construction of engineering facts’. As she shows, the forms of engineering knowledge that largely structure the development of energy technologies rely on an epistemic operation that defines ‘problems’ to be solved through a kind of radical delimitation, something she calls ‘selective blindness’. As Abram argues, the pervasiveness of selective blindness as an epistemic mode both ‘enables and limits[s] the thinkability of different [energy] horizons’.

And, finally, Jessica Smith likewise interrogates the forms of knowledge that structure energy transitions, in this case those at the centre of a massive energy infrastructure programme in the US known as Justice40. Conceived as part of the Biden administration’s national energy policy, the programme includes lofty ambitions around integrating a clean energy transition with wider goals of justice, reparation, and community well-being. However, as Smith shows, the programme relies on forms of social and environmental measurement that assume commensurability across vastly divergent categories and experiences. As she argues, the actual incommensurabilities within and across everyday practice and local histories undermine Justice40’s broader objectives, and, in the process, reinforce existing energy vulnerabilities within communities.

## Conclusion: Energy transitions beyond the socio-technical

In rejecting the colonial framing of resource extraction as a matter of harnessing the power of fossil *fuels*, Métis scholar Zoe Todd instead re-frames what is extracted from Indigenous homelands in Alberta, Canada, as fossil *kin* (2022: 4). Todd argues that the fossilized beings in Alberta’s petro-deposits have long been weaponized by the settler-colonial Canadian state. These fossil kin are extracted and transformed into commodities and pollution that disrupt reciprocal relations between human and non-human beings in Alberta’s watersheds, landscapes, and atmospheres. Relatedly, Leo Coleman has argued that energy transitions must be understood well beyond the socio-technical and political frameworks that both dominate energy policy making and justify the colonialist interpretations of ‘energy’ and ‘fuel’ decried by Indigenous scholars like Todd. Instead, Coleman suggests that a more radical perspective would expand the frame of reference to encompass the ‘counterflows and unexpected bonds of solidarity – with other people, but also with sources of energy that may exceed all existing political geographies’ (2021: 193; see also Coleman, 2017).

Perhaps this is the widest possible reading of this special issue on *Contesting Transitions*. In drawing attention to the diverse, even incommensurable, moral worlds, places, scales, histories, and forms of knowledge that shape actually existing energy transitions around the world, our collection of articles also signals a number of structural contradictions at the core of *the* global energy transition and its planetary ambitions and emergent modes of earthly praxis (Whitington and Oguz, 2023). These contradictions can be found across a broad range, from the ways in which images of ‘transition’ seek to maintain, as much as possible, both current energy infrastructure and consumption patterns, to the fact that the supposed transformative shift to sustainable technologies and ways of life is taking place in terms of the same (if ‘late’) capitalist political economy that has driven Anthropocenic harm for centuries.

Yet, beyond the fact that recent studies of supposedly green energy technologies make clear that ‘extracting the future’ (Goodale, forthcoming) from the carbon present is a non-starter, at least if paradigm-altering change is the real objective, there is also a wide gap between current energy policies at international and regional levels, and the real possibility for energy, climate, planetary, and other new figures of justice. Although the articles in this special issue do not claim to point towards ways of resolving these contradictions, or bridging these gaps, they do underscore both the urgency of the stakes involved and the indispensable place for a critical anthropology in mediating and clarify these stakes.
